# 3,8-Dimethyl-4-oxo-3,4-dihydro­quinazoline-6-carbonitrile

**DOI:** 10.1107/S1600536812022088

**Published:** 2012-05-31

**Authors:** Hai-Yan Tian, Jian-Cong Bi, Hong-Xia Zhang, Juan Zhang

**Affiliations:** a Key Laboratory of Green Chemical Engineering and Technology of Colleges of Heilongjiang Province, College of Chemical and Environmental Engineering, Harbin University of Science and Technology, Harbin 150040, People’s Republic of China

## Abstract

In the title compound, C_11_H_9_N_3_O, the quinazoline unit is almost planar, with a mean deviation of 0.006 (1) Å from the least-squares plane defined by the ten constituent atoms. In the crystal, mol­ecules are linked by weak C—H⋯N hydrogen bonds.

## Related literature

For the synthesis of the title compound, see: Shapiro *et al.* (2006 ▶).
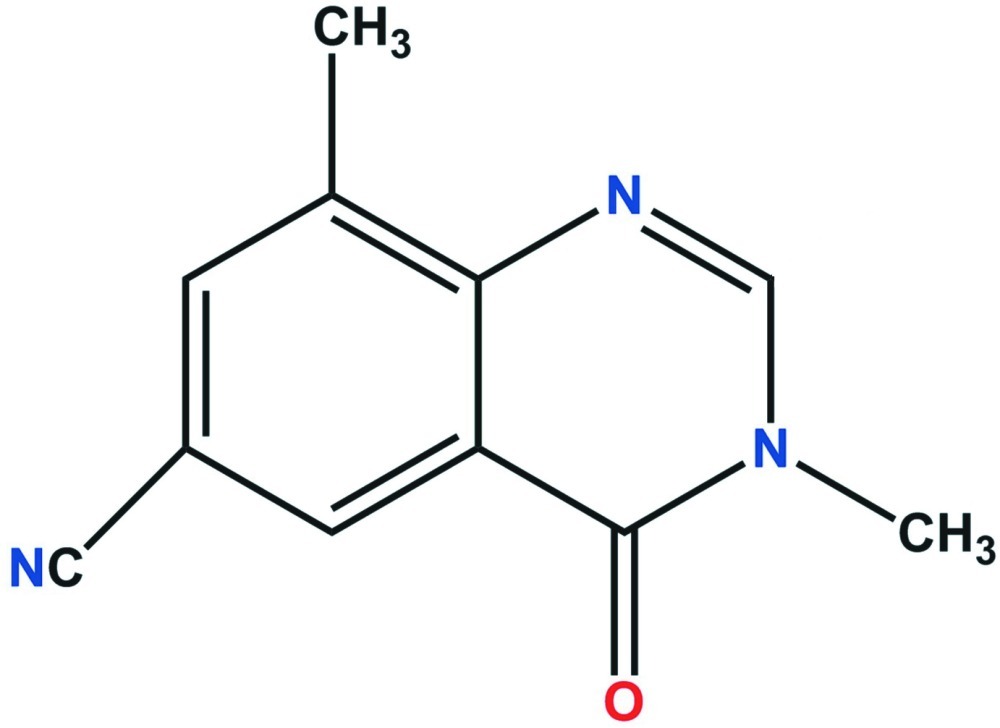


## Experimental

### 

#### Crystal data

C_11_H_9_N_3_O
*M* *_r_* = 199.21
Orthorhombic, 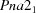

*a* = 7.0700 (14) Å
*b* = 13.441 (3) Å
*c* = 10.156 (2) Å
*V* = 965.1 (3) Å^3^
*Z* = 4
Mo *K*α radiation
μ = 0.09 mm^−1^
*T* = 293 K
0.45 × 0.30 × 0.25 mm

#### Data collection

Rigaku R-AXIS RAPID diffractometer
Absorption correction: multi-scan (*ABSCOR*; Higashi, 1995 ▶) *T*_min_ = 0.960, *T*_max_ = 0.977
8874 measured reflections
1162 independent reflections
1054 reflections with *I* > 2σ(*I*)
*R*_int_ = 0.024

#### Refinement

*R*[*F* ^2^ > 2σ(*F* ^2^)] = 0.035
*wR*(*F* ^2^) = 0.095
*S* = 1.07
1162 reflections
138 parameters
1 restraint
H-atom parameters constrained
Δρ_max_ = 0.22 e Å^−3^
Δρ_min_ = −0.20 e Å^−3^

### 

Data collection: *RAPID-AUTO* (Rigaku, 1998 ▶); cell refinement: *RAPID-AUTO*; data reduction: *CrystalClear* (Rigaku/MSC, 2002 ▶); program(s) used to solve structure: *SHELXS97* (Sheldrick, 2008 ▶); program(s) used to refine structure: *SHELXL97* (Sheldrick, 2008 ▶); molecular graphics: *SHELXTL* (Sheldrick, 2008 ▶); software used to prepare material for publication: *SHELXL97*.

## Supplementary Material

Crystal structure: contains datablock(s) I, global. DOI: 10.1107/S1600536812022088/lx2242sup1.cif

Structure factors: contains datablock(s) I. DOI: 10.1107/S1600536812022088/lx2242Isup2.hkl

Supplementary material file. DOI: 10.1107/S1600536812022088/lx2242Isup3.cml

Additional supplementary materials:  crystallographic information; 3D view; checkCIF report

## Figures and Tables

**Table 1:** Hydrogen-bond geometry (Å, °)

*D*—H⋯*A*	*D*—H	H⋯*A*	*D*⋯*A*	*D*—H⋯*A*
C8—H8⋯N1^i^	0.93	2.58	3.431 (3)	152

Symmetry code: (i) 
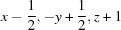
.

**Fig. 1.**
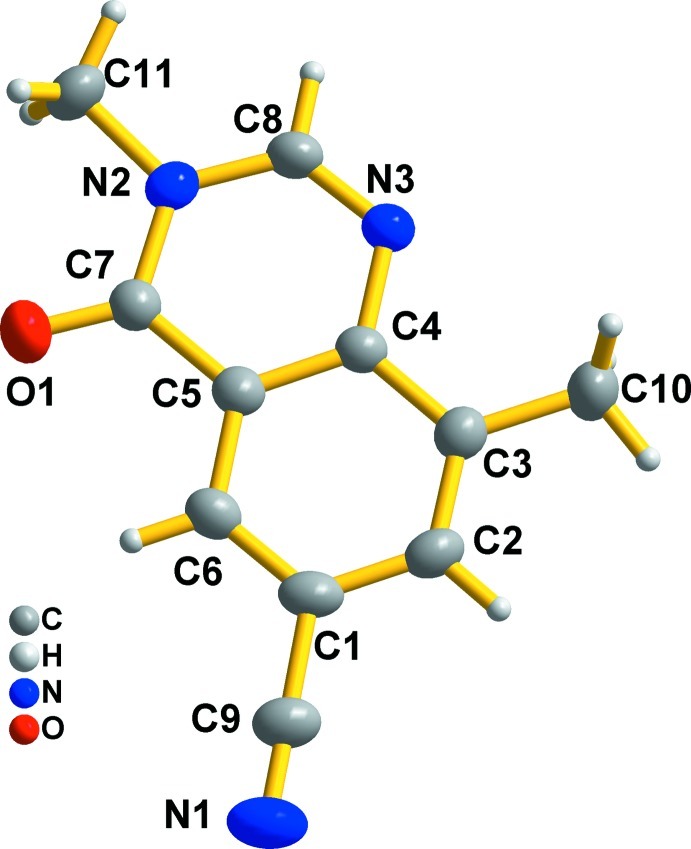
The molecular structure of the title compound with the atom numbering scheme. Displacement ellipsoids are drawn at the 50% probability level. H atoms are presented as a small spheres of arbitrary radius.

## supplementary crystallographic information

### Comment

Anthranilic diamide compounds are known as new broad spectrum pesticides, which are developed and produced by E.I.Du Pont Company. The title compound is an important intermediate of this kind of pesticides. Herein, we report the crystal structure of the title compound. The title compound crystallizes as the non-centrosymmetric space group *P*_na21_ in spite of having no asymmetric C atoms.

In the title molecule (Fig. 1), the quinazoline unit is almost planar, with a mean deviation of 0.006 (1) Å from the least-squares plane defined by the ten constituent atoms. In the crystal structure, molecules are connected by weak intermolecular C—H···N hydrogen bonds (Table 1).

### Experimental

The title compound was prepared by the reaction of N-methyl-2-amino-5-cyano-3-methylbenzamide and NaCN in DMSO under reflux conditions (Shapiro *et al.*, 2006). Single crystals suitable for X-ray diffraction were obtained by recrystallization of the title compound from ethyl acetate.

### Refinement

All the Friedel pairs were merged. H atoms bound to C atoms were placed in calculated positions and treated as riding on their parent atoms, with C—H = 0.93 / 0.96 Å (aromatic / methyl), and with *U*_iso_(H) = 1.2 / 1.5 *U*_eq_(C) (aromatic / methyl). The positions of methyl hydrogens were optimized rotationally.

### Crystal data

**Table:**

C_11_H_9_N_3_O	*F*(000) = 416
*M**_r_* = 199.21	*D*_x_ = 1.371 Mg m^−^^3^
Orthorhombic, *P**n**a*2_1_	Mo *K*α radiation, λ = 0.71073 Å
Hall symbol: P 2c -2n	Cell parameters from 8275 reflections
*a* = 7.0700 (14) Å	θ = 3.0–27.5°
*b* = 13.441 (3) Å	µ = 0.09 mm^−^^1^
*c* = 10.156 (2) Å	*T* = 293 K
*V* = 965.1 (3) Å^3^	Blcok, colorless
*Z* = 4	0.45 × 0.30 × 0.25 mm

### Data collection

**Table:**

Rigaku R-AXIS RAPID diffractometer	1162 independent reflections
Radiation source: fine-focus sealed tube	1054 reflections with *I* > 2σ(*I*)
Graphite monochromator	*R*_int_ = 0.024
ω scan	θ_max_ = 27.5°, θ_min_ = 3.0°
Absorption correction: multi-scan (*ABSCOR*; Higashi, 1995)	*h* = −9→8
*T*_min_ = 0.960, *T*_max_ = 0.977	*k* = −17→17
8874 measured reflections	*l* = −13→13

### Refinement

**Table:**

Refinement on *F*^2^	Primary atom site location: structure-invariant direct methods
Least-squares matrix: full	Secondary atom site location: difference Fourier map
*R*[*F*^2^ > 2σ(*F*^2^)] = 0.035	Hydrogen site location: difference Fourier map
*wR*(*F*^2^) = 0.095	H-atom parameters constrained
*S* = 1.07	*w* = 1/[σ^2^(*F*_o_^2^) + (0.0736*P*)^2^ + 0.0037*P*] where *P* = (*F*_o_^2^ + 2*F*_c_^2^)/3
1162 reflections	(Δ/σ)_max_ < 0.001
138 parameters	Δρ_max_ = 0.22 e Å^−^^3^
1 restraint	Δρ_min_ = −0.20 e Å^−^^3^

### Special details

**Table:**

Geometry. All esds (except the esd in the dihedral angle between two l.s. planes) are estimated using the full covariance matrix. The cell esds are taken into account individually in the estimation of esds in distances, angles and torsion angles; correlations between esds in cell parameters are only used when they are defined by crystal symmetry. An approximate (isotropic) treatment of cell esds is used for estimating esds involving l.s. planes.
Refinement. Refinement of F^2^ against ALL reflections. The weighted R-factor wR and goodness of fit S are based on F^2^, conventional R-factors R are based on F, with F set to zero for negative F^2^. The threshold expression of F^2^ > 2sigma(F^2^) is used only for calculating R-factors(gt) etc. and is not relevant to the choice of reflections for refinement. R-factors based on F^2^ are statistically about twice as large as those based on F, and R- factors based on ALL data will be even larger.

### Fractional atomic coordinates and isotropic or equivalent isotropic displacement parameters (Å^2^)

**Table:**

	*x*	*y*	*z*	*U*_iso_*/*U*_eq_	
C1	0.1413 (2)	0.27920 (15)	−0.02167 (19)	0.0378 (4)	
C2	0.1206 (3)	0.37812 (15)	0.02217 (19)	0.0396 (4)	
H2	0.1394	0.4300	−0.0370	0.048*	
C3	0.0734 (2)	0.39952 (12)	0.1495 (2)	0.0370 (4)	
C4	0.0470 (2)	0.31970 (13)	0.23889 (19)	0.0310 (3)	
C5	0.0689 (2)	0.22115 (12)	0.19457 (18)	0.0323 (4)	
C6	0.1155 (2)	0.20073 (14)	0.06421 (19)	0.0370 (4)	
H6	0.1290	0.1354	0.0355	0.044*	
C7	0.0396 (2)	0.13796 (13)	0.28618 (19)	0.0359 (4)	
C8	−0.0271 (3)	0.26620 (13)	0.44539 (19)	0.0381 (4)	
H8	−0.0603	0.2803	0.5320	0.046*	
C9	0.1888 (3)	0.25977 (17)	−0.1578 (2)	0.0446 (5)	
C10	0.0482 (4)	0.50555 (16)	0.1950 (3)	0.0545 (6)	
H10A	0.0682	0.5499	0.1222	0.082*	
H10B	0.1382	0.5200	0.2632	0.082*	
H10C	−0.0776	0.5143	0.2285	0.082*	
C11	−0.0442 (4)	0.09042 (15)	0.5119 (2)	0.0525 (6)	
H11A	−0.0593	0.1209	0.5968	0.079*	
H11B	0.0612	0.0454	0.5142	0.079*	
H11C	−0.1570	0.0544	0.4897	0.079*	
N1	0.2256 (3)	0.24416 (16)	−0.2643 (2)	0.0609 (5)	
N2	−0.0095 (2)	0.16788 (11)	0.41248 (17)	0.0370 (4)	
N3	−0.0021 (2)	0.34132 (12)	0.36812 (16)	0.0375 (4)	
O1	0.0553 (2)	0.05017 (10)	0.25774 (18)	0.0547 (4)	

### Atomic displacement parameters (Å^2^)

**Table:**

	*U*^11^	*U*^22^	*U*^33^	*U*^12^	*U*^13^	*U*^23^
C1	0.0338 (8)	0.0531 (10)	0.0265 (9)	−0.0022 (8)	0.0019 (7)	−0.0016 (8)
C2	0.0431 (9)	0.0448 (9)	0.0309 (9)	−0.0036 (7)	0.0018 (7)	0.0083 (7)
C3	0.0401 (8)	0.0352 (8)	0.0356 (10)	−0.0020 (7)	0.0015 (7)	0.0033 (8)
C4	0.0323 (7)	0.0332 (8)	0.0276 (8)	−0.0002 (6)	0.0006 (6)	−0.0011 (6)
C5	0.0316 (7)	0.0349 (8)	0.0304 (9)	−0.0007 (6)	−0.0001 (6)	−0.0017 (7)
C6	0.0385 (9)	0.0392 (8)	0.0332 (10)	−0.0002 (7)	0.0023 (7)	−0.0057 (7)
C7	0.0402 (8)	0.0340 (8)	0.0335 (9)	−0.0003 (7)	0.0007 (7)	−0.0009 (7)
C8	0.0461 (9)	0.0403 (9)	0.0279 (9)	0.0028 (7)	0.0042 (7)	−0.0013 (7)
C9	0.0431 (10)	0.0586 (12)	0.0321 (11)	−0.0015 (9)	0.0039 (8)	0.0013 (8)
C10	0.0849 (15)	0.0336 (9)	0.0450 (12)	0.0009 (10)	0.0096 (11)	0.0033 (8)
C11	0.0754 (13)	0.0433 (10)	0.0387 (13)	0.0026 (10)	0.0104 (10)	0.0121 (9)
N1	0.0690 (12)	0.0809 (14)	0.0329 (10)	0.0012 (10)	0.0099 (9)	−0.0037 (9)
N2	0.0441 (8)	0.0371 (8)	0.0298 (9)	0.0001 (6)	0.0016 (6)	0.0042 (6)
N3	0.0482 (8)	0.0356 (7)	0.0287 (9)	0.0012 (7)	0.0051 (6)	−0.0023 (6)
O1	0.0816 (11)	0.0328 (6)	0.0498 (10)	0.0011 (7)	0.0053 (8)	−0.0036 (6)

### Geometric parameters (Å, º)

**Table:**

C1—C6	1.381 (3)	C7—N2	1.388 (3)
C1—C2	1.410 (3)	C8—N3	1.291 (2)
C1—C9	1.447 (3)	C8—N2	1.369 (2)
C2—C3	1.366 (3)	C8—H8	0.9300
C2—H2	0.9300	C9—N1	1.132 (3)
C3—C4	1.418 (2)	C10—H10A	0.9600
C3—C10	1.508 (3)	C10—H10B	0.9600
C4—N3	1.388 (2)	C10—H10C	0.9600
C4—C5	1.408 (2)	C11—N2	1.471 (2)
C5—C6	1.392 (3)	C11—H11A	0.9600
C5—C7	1.469 (2)	C11—H11B	0.9600
C6—H6	0.9300	C11—H11C	0.9600
C7—O1	1.220 (2)		
			
C6—C1—C2	120.46 (18)	N3—C8—N2	126.47 (18)
C6—C1—C9	119.78 (19)	N3—C8—H8	116.8
C2—C1—C9	119.76 (19)	N2—C8—H8	116.8
C3—C2—C1	121.54 (17)	N1—C9—C1	179.7 (3)
C3—C2—H2	119.2	C3—C10—H10A	109.5
C1—C2—H2	119.2	C3—C10—H10B	109.5
C2—C3—C4	118.61 (17)	H10A—C10—H10B	109.5
C2—C3—C10	121.16 (18)	C3—C10—H10C	109.5
C4—C3—C10	120.23 (18)	H10A—C10—H10C	109.5
N3—C4—C5	121.79 (16)	H10B—C10—H10C	109.5
N3—C4—C3	118.67 (16)	N2—C11—H11A	109.5
C5—C4—C3	119.54 (16)	N2—C11—H11B	109.5
C6—C5—C4	121.05 (16)	H11A—C11—H11B	109.5
C6—C5—C7	119.06 (15)	N2—C11—H11C	109.5
C4—C5—C7	119.88 (16)	H11A—C11—H11C	109.5
C1—C6—C5	118.80 (16)	H11B—C11—H11C	109.5
C1—C6—H6	120.6	C8—N2—C7	121.87 (16)
C5—C6—H6	120.6	C8—N2—C11	120.03 (18)
O1—C7—N2	121.44 (18)	C7—N2—C11	118.10 (16)
O1—C7—C5	125.00 (19)	C8—N3—C4	116.43 (16)
N2—C7—C5	113.56 (15)		

### Hydrogen-bond geometry (Å, º)

**Table:**

*D*—H···*A*	*D*—H	H···*A*	*D*···*A*	*D*—H···*A*
C8—H8···N1^i^	0.93	2.58	3.431 (3)	152

Symmetry code: (i) *x*−1/2, −*y*+1/2, *z*+1.
